# Mental pain and suicide risk: implications for neuroimaging

**DOI:** 10.1192/j.eurpsy.2023.137

**Published:** 2023-07-19

**Authors:** M. Pompili

**Affiliations:** Sapienza University of Rome, Rome, Italy

## Abstract

**Abstract:**

Campaigns have been launched to make sense of what makes a specific individual suicidal. We know that suicidal individuals give definite warning signs, mainly from their ambivalence about ending their own lives. Classical suicidology posited that the suicidal individual experiences unbearable psychological pain (*psychache*) or suffering and that suicide might be, at least in part, an attempt to escape from this suffering, emphasizing that suicide is not a movement toward death but rather an escape from unbearable emotion and unendurable or unacceptable anguish. Suicide occurs when that individual deems the psychache to be unbearable. Neuroimaging studies demonstrated that such emotional pain shares the same neuroanatomical circuit of somatic pain. Furthermore, concepts related to death, failure, or other unfortunate circumstances activate specific cerebral areas in a suicidal individual compared to a non-suicidal subject. The author conducted a sizeable clinical investigation on mental pain related to psychiatric disorders and suicide risk. Implications for further research are discussed during the presentation.

**Disclosure of Interest:**

M. Pompili Consultant of: Janssen, Lundbeck, Recordati, MSD, Speakers bureau of: Janssen, Lundbeck, Angelini Pharma, Pfizer

